# Gabapentin Attenuates Ocular Inflammation: *In vitro* and *In vivo* Studies

**DOI:** 10.3389/fphar.2017.00173

**Published:** 2017-04-04

**Authors:** Carmelina D. Anfuso, Melania Olivieri, Annamaria Fidilio, Gabriella Lupo, Dario Rusciano, Salvatore Pezzino, Caterina Gagliano, Filippo Drago, Claudio Bucolo

**Affiliations:** ^1^Department of Biomedical and Biotechnological Sciences, School of Medicine, University of CataniaCatania, Italy; ^2^Sooft Italia S.p.A.Rome, Italy; ^3^Bioos s.r.l.Catania, Italy; ^4^Eye Clinic, Santa Marta Hospital, University of CataniaCatania, Italy

**Keywords:** gabapentin, corneal cells, endotoxin-induced uveitis, ocular inflammation, TNF-α

## Abstract

To investigate the effects of gabapentin, a structural analog of γ-amino butyric acid (GABA), on the inflammatory response of lipopolysaccharide (LPS)-stimulated rabbit corneal cells (SIRC) and on endotoxin-induced uveitis (EIU) in rabbits. We investigated the LPS-induced expression of several inflammatory mediators, such as TNF-α, IL-1β, cPLA_2_, COX-2, and PGE_2_ in the SIRC cells with or without gabapentin treatment. Gabapentin treatment significantly (*p* < 0.05) attenuated cytokines production, cPLA_2_ activation, COX-2 expression, and PGE_2_ levels in SIRC. EIU was induced by an intraocular injection of 0.1 μg of LPS in albino rabbit eye. After 7 and 24 h from LPS injection clinical signs of ocular inflammation were examined by slit lamp with or without topical treatment of 0.5% gabapentin. Tears, aqueous, cornea, conjunctiva, and iris-ciliary body were collected and inflammatory biomarkers assessed. Topical treatment with gabapentin significantly (*p* < 0.05) reduced clinical signs and biomarkers of inflammation compared with the LPS group both at 7 and 24 h. In conclusion, the results generated in the present study suggest that ophthalmic formulation based on gabapentin may be useful in the treatment of inflammatory conditions associated to ocular pain such as uveitis, and that clinical studies to evaluate this possibility may be warranted.

## Introduction

Gabapentin, a structural analog of γ-amino butyric acid (GABA), targeting α_2_δ_1_ subunit of voltage-sensitive calcium channels, has therapeutic effect for neurological and psychiatric disorders such as epilepsy, anxiety, and neuropathic pain. This latter is also present in the eye and has been reported in patients affected by dry eye disease or diabetes or Sjögren syndrome (Rosenthal et al., 2009). Further, neuropathic ocular pain can be elicited by UV exposure or chemical insults. The effect of gabapentin on pain as recently linked to the anti-inflammatory action of the drug. It has been demonstrated (Lee et al., 2013) that gabapentin is able to reduce pro-inflammatory mediators (e.g., TNF-α, IL-1β, and IL-6) and up-regulates anti-inflammatory cytokine IL-10 in a rat model of neuropathic pain. More recently (Dias et al., 2014) it has been showed that gabapentin reverses inflammatory process in well-known acute mouse models of inflammation.

Inflammation is a non-specific response to injury that includes a variety of functional and molecular mediators, including recruitment and/or activation of inflammatory cells and release of inflammatory mediators such as cytokines (e.g., TNF-α), interleukins (e.g., IL-1β, IL-6), enzymes [e.g., cyclooxygenases (COXs)], and prostaglandins (PGs) (e.g., PGE_2_). These latter are synthesized by COXs, bifunctional enzymes which contain both cyclooxygenase and peroxidase activity and exist as distinct isoforms referred to as COX-1 and COX-2 (Smith et al., 2000) starting from arachidonic acid (AA), in turn hydrolyzed by phospholipases A_2_ (PLA_2_s). Moreover, AA plays a key role in inflammation and neurodegenerative disorders (Sun et al., 2004). Among the three major classes of PLA_2_s (secretory, calcium-independent and calcium-dependent) in the mammalians, the group IV calcium-dependent cytosolic PLA_2_α (cPLA_2_α) has received the most attention because of its expression in all mammalian cells and its active participation in cell metabolism (Sun et al., 2014). Following the Ca^++^binding to its C-2 domain, cPLA_2_ undergoes a number of post-translational modifications, such as phosphorylation on Ser-505, -727, and -515 and *S*-nitrosylation after the NO-interaction (Linkous and Yazlovitskaya, 2010). The aim of the study was to investigate the effects of gabapentin on ocular inflammatory models using lipopolysaccharide (LPS)-induced damage both *in vitro* and *in vivo*. In particular, we used rabbit corneal cells [Seruminstitute Rabbit Cornea (SIRC)] and a rabbit model of uveitis. Endotoxin-induced uveitis (EIU) represents a valuable experimental model of acute ocular inflammation characterized by release of several inflammatory biomarkers. We investigated the effect of gabapentin both in corneal cells challenging with LPS and in EIU assessing the levels of TNF-α, IL-6, IL-1β, and PGE_2_.

## Materials and Methods

### Materials

Gabapentin was purchased from Santa Cruz Biotechnology (Santa Cruz, CA, USA). LPS (L2880) from *Escherichia coli* O127:B8 were obtained from Sigma–Aldrich Chemical Co. (St Louis, MO, USA). Anti-rabbit IL-1β and anti-mouse TNF-α antibodies were purchased from Abcam Inc. (Cambridge, MA, USA). Anti-rabbit p-cPLA_2_, cPLA_2_, COX-2 antibodies were purchased from Cell Signaling Technology (CST).

### Cell Cultures

Statens Seruminstitut rabbit corneal (SIRC) epithelial cells (ATCC CCL-60) were cultured in minimum essential medium with Earle’s salts, L-glutamine, and non-essential amino acids supplemented [Eagle’s Minimum Essential Medium (ATCC^®^ 30-2003^TM^] with 10% activated fetal bovine serum (FBS, 10108-165, GIBCO), incubated at 37°C in a humidified atmosphere of 5% CO_2_. SIRC cells were seeded into 6-wells plates for 24 h before the experiment at a density of 1.2 × 10^5^ cells/well in 2.0 ml of medium. Culture medium was exchanged every other day, and cultures were maintained until sub-confluence. For all experiments, cells were pre-incubated in serum-free medium (SFM) for 1 h with 10 μg/ml gabapentin (this concentration was chosen based on cell viability assay) and then, the inflammatory stimulus was induced with LPS (1 μg/ml) for different times. In addition, in order to examine the gabapentin effect on baseline cytokines level, SIRC cells were incubated with the drug alone without LPS.

### Analysis of Cell Viability

The MTT assay was employed to assess rabbit corneal cell viability after treatment with gabapentin or LPS. Cells were seeded in 96-well plates at a density 2 × 10^4^ cells per well and incubated overnight at 37°C before experiment. Subsequently, different concentrations of gabapentin (5, 10, 100, 1000 μg/ml) were added to each well except the well with control solution, for 24 and 48 h. In a second set of experiments was also assayed the toxicity of LPS (1–10–100 μg/ml) after 24 h. After incubation with the substances, 10 μl MTT [3-(4,5-dimethylthiazol-2-yl)2,5-diphenyltetrazolium bromide] reagent (5 mg/ml) was added to each well and the plates were incubated for 3 h at 37°C. The formazan crystals were extracted with 100 μl DMSO and plates were shaken for 10 min. The absorbance was measured at 570 nm with plate reader (Biotek Instruments, Elx-800). Cell viability was calculated as a percentage of the control.

### Western Blot Analysis

Protein was extracted from SIRC cells as follows: cells were washed with PBS (pH 7.4), centrifuged at 1000 × *g* for 3 min, suspended in protein extraction buffer and incubated on ice for 30 min. After sonication, extract was centrifuged at 8,000 × *g* for 15 min. Protein samples (30 μg/lane) were subjected to SDS-PAGE and, after transfer to nitrocellulose membranes, were incubated as described previously (Lupo et al., 2007; Scuderi et al., 2008) with antibody against TNF-α, IL-1β, p-cPLA2, cPLA2, COX-2, β-actin overnight at 4°C followed by incubation with horseradish peroxidase conjugated secondary antibody, goat anti-rabbit IgG for IL-1β, p-cPLA2, cPLA2, COX-2 and goat anti-mouse IgG for TNF-α and β-actin. After washing, with TBS-T, protein expression was visualized with the Super Signal West Pico Chemiluminescence detection system (Thermo Scientific, Rockford, IL, USA). β-actin served as the loading control. Bands were analyzed using Image J software (Version1.43, Broken Symmetry Software, Bethesda, MD, USA).

### PGE_2_ Measurement

PGE_2_ was measured in culture medium using a competitive binding ELISA, according to the manufacturer’s instructions (Abcam Inc., Cambridge, MA, USA). In particular, the SFM was harvested after incubation of SIRC with gabapentin and LPS (as described in experimental design) for 24 h. The amount of PGE_2_ was extrapolated from a standard curve (according the manufacturer’s instructions). All experiments were performed in triplicate.

### Endotoxin-Induced Uveitis (EIU) Model

Male New Zealand albino rabbits weighing 2–2.5 kg (Harlan, Italy) were used. Animals were housed in single cage upon arrival in the facilities (in a light and temperature controlled room) with tap water and standard chow provided *ad libitum*. Animal procedures were approved by the Institutional Animal Care and Use Committee (IACUC) of the University of Catania, and conformed to the Association for Research in Vision and Ophthalmology (ARVO) resolution on the use of animals in research. Uveitis was induced by an intravitreal injection of LPS (0.1 μg/10 μl of *E. coli* 0111:B4; Sigma–Aldrich, Milan, Italy). Before LPS injection, rabbits were anesthetized by intravenous injection of 5 mg/kg Zoletil^®^ (2.5 mg/kg tiletamine HCl and 2.5 mg/kg zolazepam HCl; Virbac, Milan, Italy) and one drop of local anaesthetic (Novesina^®^, Novartis, Origgio, Italy) was administered to the eye. An ophthalmic formulation of 0.5% gabapentin was prepared in an isotonic buffered solution (pH 7.0; 298 mOsm) and a multiple treatment (50 μl/instillation) was carried out (one instillation 30 min before LPS and four treatments after LPS). The dose used in the present study was chosen based on preliminary dose-ranging study (data not shown). Seven or 24 h after LPS injection tears were obtained with glass capillary tubes (Behring Diagnostics, Marburg, Germany). After that, the animals were killed (Tanax^®^; Intervet, Milan, Italy) and aqueous, conjunctiva, cornea, and iris-ciliary body collected. Care was taken to obtained tear samples avoiding stimulated tear production. Ten microliters of tears were collected from each eye and stored at -80°C until analysis.

### Clinical Score and Aqueous Protein Levels

The clinical signs of ocular inflammation were examined by slit lamp (Sbisà, Firenze, Italy) and were graded on a scale of 0–4, according to the scoring system described by Ruiz-Moreno et al. (1992). Briefly the score was: 0 = no inflammatory reaction; 1 = discrete inflammatory reaction; 2 = moderate dilation of the iris and conjunctival vessels; 3 = intense iridal hyperemia, with flare in the anterior chamber; 4 = the same clinical signs as grade 3 plus the presence of fibrinoid exudation in the pupillary area, with intense flare in the anterior chamber. Clinical signs assessment was performed 15 m before the 7th and 24th h from intravitreal injection of LPS. Aqueous samples were collected from both eyes with a 30-gauge needle and protein levels assessed by a bicinchoninic acid (BCA) protein assay kit (Beyotime Institute of Biotechnology, Jiangsu, China).

### Biomarkers Assessment

Ocular tissues (cornea, conjunctiva. and iris-ciliary body) were cut and lysed in 500 μL of tissue extraction reagent containing protease inhibitors (Invitrogen) with ULTRA-TURRAX. The samples were sonicated on ice and centrifuged twice (15,000 × *g* at 4°C, 20 min). The supernatant was assayed for levels of pro-inflammatory cytokines with commercial ELISA kits for TNF-α, IL-6, IL-1β (Quantikine kit; life technologies); before ELISA assay, total protein content in the ocular tissues was measured using the BCA protein assay kit. The ratio of cytokine to total protein (pg/mg) was calculated. All estimations were performed in duplicate. The TNF-α level was evaluated in tears with commercial ELISA kits (Quantikine kit; life technologies) at 7 and 24 h after injection of LPS; for the analysis were used 5 μL of the sample and values were expressed as pg/mL. In the aqueous humor, the TNF-α level was evaluated at 7 h and 24 h after injection of LPS with commercial ELISA kits (Quantikine kit; MybioSource) using 50 μL of the sample and values were expressed as pg/mL. PGE_2_ levels in aqueous humor samples were measured using an ELISA kit (Quantikine kit, Abcam) at 7 and 24 h after injection of LPS. The values were expressed as pg/mL according to the instruction manual.

### Statistical Analysis

Data are presented as mean ± SD. A statistical analysis was performed with GraphPad Prism (GraphPad Software, Inc., San Diego, CA, USA). One-way ANOVA, followed by *Tukey’s test* was applied for parametric data; *Kruskal–Wallis test*, followed by *Mann–Whitney test* was performed for non-parametric data. A *P*-value less than 0.05 was considered statistically significant.

## Results

### Cell Viability

**FIGURE 1:**
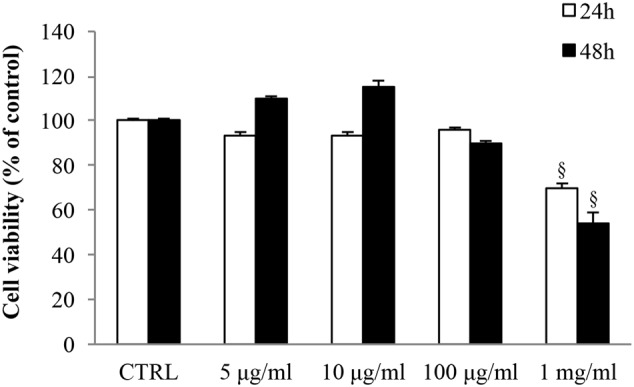
**Effects of gabapentin on corneal cells viability.** SIRC were seeded in 96-well plates at a density of 2 × 10^4^ cells per well and incubated overnight at 37°C before experiment. Subsequently, different doses of gabapentin were added to each well for 24 and 48 h, as described in section “Materials and Methods.” Values represent mean ± SD, ^x^*p* < 0.01 *vs.* CTRL. One-way ANOVA, followed by *Tukey’s test.*

MTT assays were carried out in order to assess the effect of gabapentin on SIRC viability (**Figure 1**). 5, 10, and 100 μg/ml gabapentin did not affect cell viability, either at 24 h or at 48 h. Gabapentin at 1 mg/ml concentration caused a decrease in viability by 30 and 46% at 24 and 48 h, respectively. The data were confirmed by Trypan blue staining (data not shown). Based on these results, 10 μg/ml has been chosen for all *in vitro* experiments.

### Effects of Gabapentin on LPS-induced TNF-α and IL-1β Protein Expression

**FIGURE 2:**
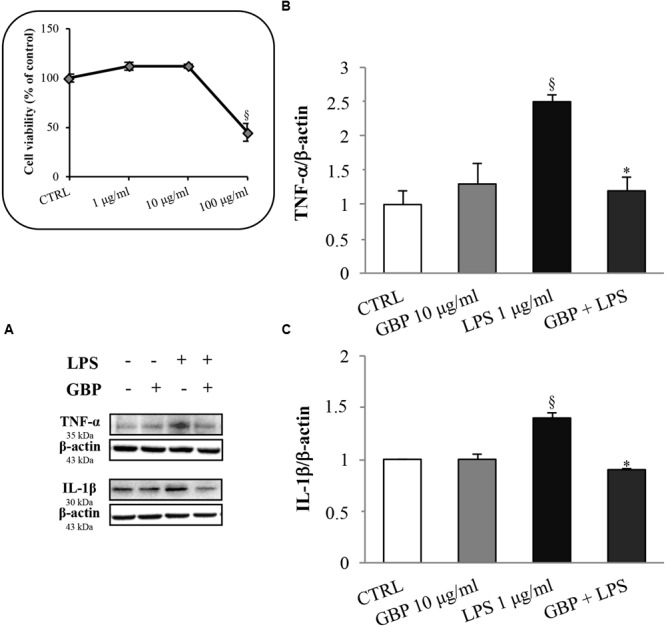
**Effects of gabapentin on cytokines production by lipopolysaccharide (LPS) in corneal cells.** SIRC were pre-incubated in serum-free medium (SFM) for 1 h with gabapentin (10 μg/ml), and the inflammatory stimulus was induced with LPS (1 μg/ml) for 24 h. Lysates were prepared for Western blot analysis as described in section “Materials and Methods.” **BOX**: Effect of LPS on SIRC viability after 24 h incubation; for the subsequent experiments was chosen the 1 μg/ml not toxic dose. **(A)** Representative immunoblot images of TNF-α, IL-1β, and β-actin following LPS treatment with or without gabapentin. **(B,C)** Densitometric analysis of TNF-α and IL-1β protein levels after adjusting for β-actin band intensity. Values represent mean ± SD, ^∗^*p* < 0.05 *vs.* LPS, ^x^*p* < 0.01 *vs.* CTRL. One-way ANOVA, followed by *Tukey’s test*.

Seruminstitute Rabbit Cornea were treated with 1 μg/ml LPS to induce an inflammatory response (**Figure 2**, see the dose-response MTT viability curve into the box in the left of the figure). LPS increased TNF-α and IL-1β expression by 2.1- and 1.4-fold, respectively (**Figure 2**). This effect was significantly attenuated by gabapentin treatment (by 52 and 36% for TNF-α and IL-1β, respectively). These data demonstrate a positive effect of gabapentin to counteract inflammatory cytokines elicited by LPS. Gabapentin (10 μg/ml) alone did not elicited expression of TNF-α and IL-1β.

### PLA_2_ Protein Expression/Activation

**FIGURE 3:**
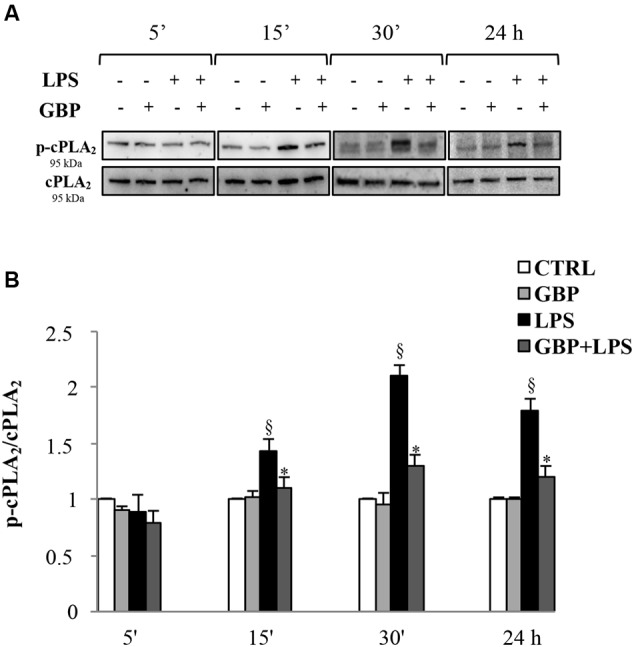
**Effects of gabapentin on cPLA_2_ activation by LPS in corneal cells.** SIRC were pre-incubated in SFM for 1 h with gabapentin (10 μg/ml) and the inflammatory stimulus was induced with LPS (1 μg/ml) for the different time points reported in figure. Lysated were prepared for Western blot analysis, as described in section “Materials and Methods.” **(A)** Representative immunoblot images of phospho-cPLA_2_ (p-cPLA_2_) and total-cPLA_2_ after LPS treatment with or without gabapentin. **(B)** Phospho-cPLA_2_/cPLA_2_ band intensity ratios of protein levels. Values represent mean ± SD, ^∗^*p* < 0.05 *vs.* LPS, ^x^*p* < 0.01 *vs.* CTRL. One-way ANOVA, followed by *Tukey’s test*.

Western blot analyses of cPLA_2_ and phospho-cPLA_2_ in SIRC lysates from LPS with or without gabapentin in time-course experiments (5, 15, and 30 min, and 24 h) are reported in **Figure 3**. Immunoblots revealed the cPLA_2_ total protein expression was almost comparable in all samples within the same slot of incubation, regardless of LPS or gabapentin presence (panel A). LPS caused significant increases in the expression of the phosphorylated protein (except at 5 min), that is to say the activated form of cPLA_2_ (1.44, 2.1, and 1.8 p-cPLA_2_/cPLA_2_ ratio at 15 and 30 min and 24 h, respectively) and the increase in enzyme activity (**Figure 3**). Interestingly, by 15 min onward in subsequent incubation time points, gabapentin caused a significant (*p* < 0.05) reduction of the phosphorylation levels of the protein. These data support the idea that gabapentin is able to modulate the activation of an upstream enzyme in the intracellular cascade of events that lead, through the release of AA to the synthesis of key inflammatory mediators such as eicosanoids.

### COX-2 and PGE_2_ in Corneal Cells

**FIGURE 4:**
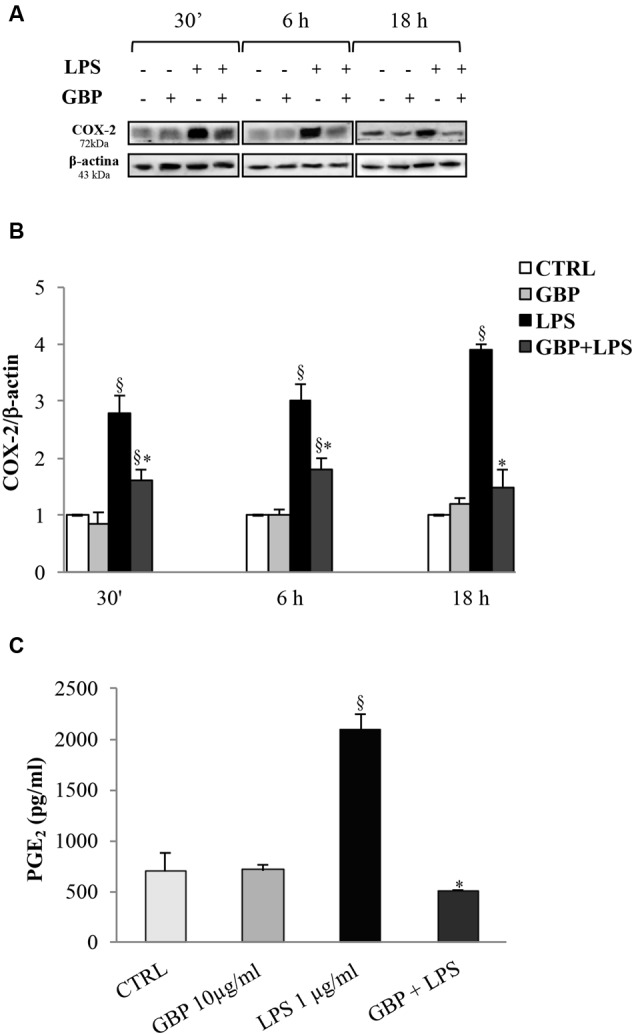
**Effects of gabapentin on COX-2 and PGE_2_ in corneal cells.** SIRC were pre-incubated in SFM for 1 h with gabapentin (10 μg/ml) and the inflammatory stimulus was induced with LPS (1 μg/ml) for the time points reported in figure. Lysated were prepared for Western blot analysis, as described in section “Materials and Methods.” In a separate set of experiments, the supernatants were collected to assess PGE_2_ after 24 h, as described in section “Materials and Methods.” Representative immunoblot images of COX-2 after LPS exposure with or without gabapentin **(A)**. Densitometric analysis of COX-2 protein level after adjusting for β-actin band intensity **(B)**. ELISA analysis of PGE_2_ levels after 24 h of LPS exposure with or without gabapentin **(C)**. Values represent mean ± SD, ^∗^*p* < 0.05 *vs.* LPS, ^x^*p* < 0.01 *vs.* CTRL. One-way ANOVA, followed by *Tukey’s test*.

Cyclooxygenases-2 expression in SIRC (**Figures 4A,B**) after 30 min, 6 and 18 h from LPS was evaluated (no changes in COX-1 protein expression were observed; data not shown). Conversely, LPS-stimulated SIRC significantly expressed inducible COX-2 total protein by 2.8-, 3.0-, and 3.9-fold at 30 min, 6 and 18 h, respectively, compared to control (no LPS) (**Figure 4B**). Gabapentin treatment (10 μg/ml) significantly (*p* < 0.05) reduced the LPS-conditioned SIRC COX-2 overexpression by 43% (30 min), 40% (6 h), and 62% (18 h). These data confirmed the positive effect of gabapentin to contrast the production of inflammatory enzymes such as COX-2. At this regard, we evaluated PGE_2_ production, measured in supernatants of all culture models incubated for 24 h (**Figure 4C**). Gabapentin alone in SIRC (without LPS) had no effect on PGE_2_ production. As expected, LPS-induced a significant increase in PGE_2_ production by 3.0-fold compared to control. Gabapentin treatment in LPS-stimulated SIRC caused a significant (*p* < 0.05) reduction in PGE_2_ levels.

### Clinical Score and Aqueous Protein Levels

**FIGURE 5:**
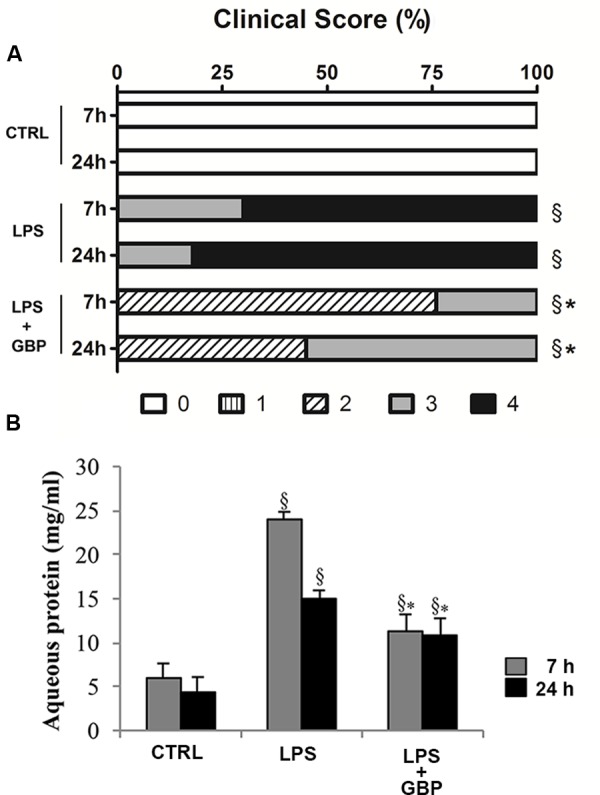
**Effects of topical gabapentin on clinical score and aqueous protein levels in experimental uveitis.** Clinical scores were evaluated at 7 and 24 h after LPS injection **(A)**, see section “Materials and Methods” for more details. Aqueous protein levels from all groups measured at 7 and 24 h after LPS injection **(B)**. Values represent mean ± SD, ^∗^*p* < 0.05 *vs.* LPS, ^x^*p* < 0.01 *vs.* CTRL. *Kruskal–Wallis* test followed by *Mann–Whitney test* **(A)**; one-way ANOVA followed by *Tukey’s test* **(B)**.

Ocular inflammation elicited by LPS caused a significant (*p* < 0.05) damage of eye’s tissues after 7 h still evident at 24 h (**Figure 5A**). Topical treatment with gabapentin significantly reduced clinical signs of inflammation compared with the LPS group both at 7 and 24 h (**Figure 5A**). Furthermore, LPS injection induced a significant (*p* < 0.01) increase of protein levels in the aqueous humor particularly after 7 h (**Figure 5B**) which is significantly (*p* < 0.05) reverted by gabapentin treatment.

### Inflammatory Biomarkers

**FIGURE 6:**
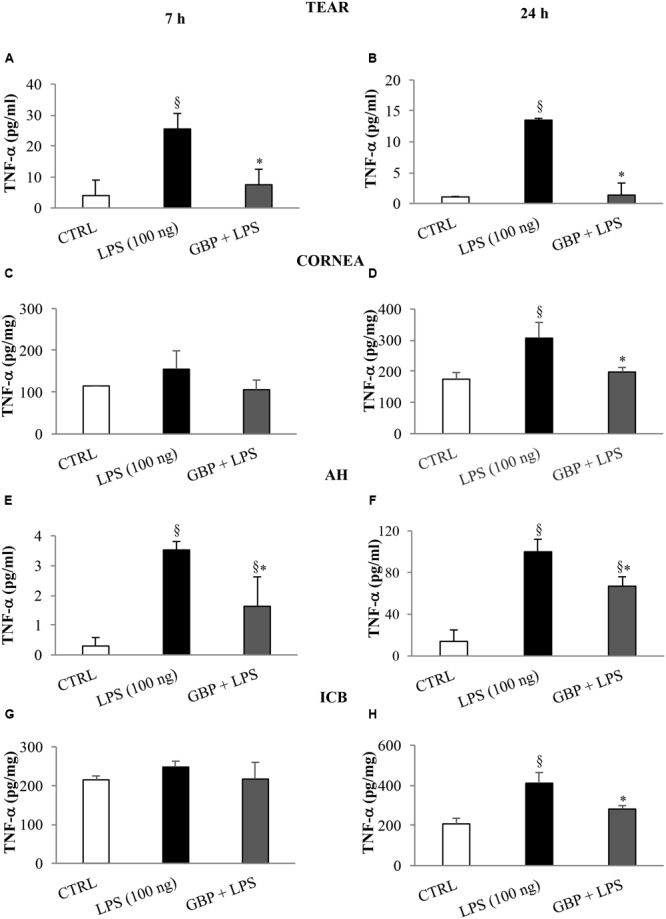
**Effects of topical gabapentin on TNF-α levels in experimental uveitis.** TNF-α levels in tears **(A,B)**, cornea **(C,D)**, aqueous humor (AH) **(E,F)**, and iris-ciliary body (ICB) **(G,H)** at 7 and 24 h after LPS injection. Values represent mean ± SD, ^∗^*p* < 0.05 *vs.* LPS, ^x^*p* < 0.01 *vs.* CTRL. One-way ANOVA, followed by *Tukey’s test*.

**FIGURE 7:**
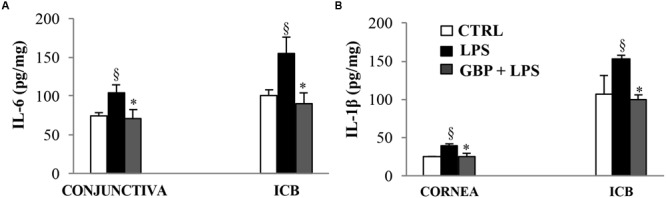
**Effects of topical gabapentin on IL-6 and IL-1β levels in experimental uveitis.** IL-6 **(A)** and IL-1β **(B)** levels in cornea, conjunctiva, and ICB **(G, H)** at 7 and 24 h, respectively, after LPS injection. Values represent mean ± SD, ^∗^*p* < 0.05 *vs.* LPS, ^x^*p* < 0.01 *vs.* CTRL. One-way ANOVA, followed by *Tukey’s test.*

**FIGURE 8:**
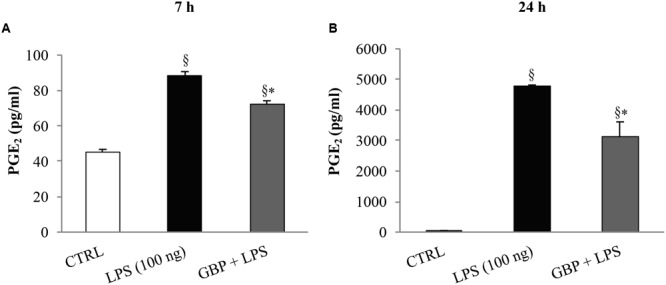
**Effect of topical gabapentin on aqueous PGE_2_ levels in experimental uveitis.** PGE_2_ levels in aqueous humor at 7 h **(A)** and 24 h **(B)** after LPS injection. Values represent mean ± SD, ^∗^*p* < 0.05 *vs.* LPS, ^x^*p* < 0.01 *vs.* CTRL. One-way ANOVA, followed by *Tukey’s test*.

At 7 and 24 h after LPS injection we observed a significant (p < 0.01 *vs*. control) increase of TNF-α levels in tears, cornea, aqueous, and iris-ciliary body that was reversed by topical treatment of gabapentin (**Figure 6**). Furthermore, the effects of topical gabapentin on IL-6 and IL-1β in ocular tissues were assessed in rabbit with EIU (**Figure 7**). Previous studies (Mo et al., 1998; Brito et al., 2006) reported that the peak of aqueous IL-1β levels in rabbit with EIU was around 18 h after endotoxin injection, and IL-6 did not change at 24 h. In accordance with these reports we observed a significant (*p* < 0.01) peak of IL-6 and IL-1β in the aqueous in rabbit with EIU at 7 and 24 h, respectively. Topical treatment with gabapentin significantly (*p* < 0.05) attenuated the release of IL-6 and IL-1β in the aqueous of rabbit with EIU (**Figure 7**). Finally, gabapentin was able to significantly reduce the PGE_2_ levels in the aqueous elicited by endotoxin injection (**Figure 8**).

## Discussion

In the present study we demonstrated that gabapentin inhibited ocular inflammation both *in vitro* and *in vivo* paradigms. In particular, gabapentin was able to counteract the inflammatory process elicited by LPS in rabbit corneal cells and rabbit’s eye.

Inflammation usually has beneficial effects on an acute basis, but can have unwanted effects if abiding chronically. Uveitis is the fourth cause of blindness in developed countries, and it represents a typical chronic ocular inflammation with a difficult management in terms of pharmacological therapy. Gabapentin is structurally related to the neurotransmitter gamma aminobutyric acid (GABA) but does not bind to the GABA receptors. Gabapentin is a drug with approved indications for epilepsy, seizures and neuropathic pain. Its mechanism of action is through binding to calcium channels and modulating the influx of calcium and thereby bestowing antiepileptic and analgesic effects. It is not clear if the anti-inflammatory effect of gabapentin is related to calcium modulation rather than other mechanisms such as stimulation of endogenous anti-oxidants like GSH, inhibition of NF-kB, block of NMDA receptor or activation of adenosine A1 receptor (Abdel-Salam and Sleem, 2009; Kim et al., 2009; Yang et al., 2012; Dias et al., 2014; Wang et al., 2014; Martins et al., 2015; Xu et al., 2017). Phosphorylation and calcium concentrations are the effectors modulating the activity of cPLA_2_ and recent studies have highlighted the role of PLAs_2_ as potential therapeutic target in inflammation and in other serious disorders, and the increase of PLA_2_ has been linked with the severity of the disease (Yarla et al., 2015). AA is released from phospholipids by the action of different isoforms of phospholipase A_2_s (PLA_2_s) and converted to PGs or leukotrienes (LTs) by the action of COXs and 5-lipoxygenase, respectively. These downstream products play key roles in governing cell migration and proliferation, as well as inflammation (Dennis et al., 2011; Anfuso et al., 2014; Lupo et al., 2014). Actually, four main groups of phospholipases are known, which include the secretory, the calcium-independent, the cytosolic and the lipoprotein-associatedphospholipases A_2_.

Release of AA has been shown to accumulate in response to ischemia in the eye (Birkle and Bazan, 1989; Remé et al., 1994). There is only a little knowledge about PLA_2_ expression in other parts of the eye, in addition to the retina (Castagnet and Giusto, 1993; Van Themsche et al., 2001; Kolko et al., 2007). cPLA has been identified in the human cornea (Landreville et al., 2004), while, in the conjunctiva, cPLA_2_ is mainly localized in the surface of the epithelium, probably participating in the protection against risks caused by mechanical wear and tear stress (Helin et al., 2008). In an *in vitro* retinoblastoma human triple culture model of angiogenesis, tumor cells induced in human retinal endothelium the increase in cPLA_2_proteinexpression (Lupo et al., 2014). As showed in the *in vitro* studies, LPS caused an increase expression of the cPLA_2_ active form and gabapentin significantly lowered the phosphorylation levels of the protein, supporting the idea that gabapentin was able to modulate the intracellular cascade of events that lead to the release of AA for the synthesis of key inflammatory eicosanoids mediators. Two main isoforms of cyclooxygenase exist, COX-1 and COX-2 (Simmons et al., 2004), being COX-3 still largely unknown (Davies et al., 2004). COX-1 is constitutively expressed in many tissues and plays a key role in the management of homeostasis. On the contrary, COX-2 is an inducible isoform and is activated in response to extracellular stimuli such as growth factors and pro-inflammatory cytokines (Simmons et al., 2004). It has been shown that cPLA_2_ and COX-2 co-localized in the perinuclear area (Pardue et al., 2003). Moreover, IL-1β and TNFα activate COX-2 and stimulate signaling pathways leading to cPLA_2_ phosphorylation and AA. At this regard, TNFα-stimulated phosphorylation of cPLA_2_involves the c-Jun and p38 MAP kinase pathways (Hernández et al., 1999). In an endotoxin-induced uveitis rodent model and in human ARPE-19 cells LPS-activated, an increasing of COX-2, IL-6, and IL-8 gene expression were found (Girol et al., 2013). Moreover, mice underwent to adverse environmental conditions showed high COX-2 and PGE synthase mRNA levels on the ocular surface (Shim et al., 2012). In accordance with this data, LPS-stimulated SIRC significantly expressed inducible COX-2 total protein and gabapentin treatment dramatically reduced the COX-2 synthesis induction. Moreover, gabapentin treatment in LPS-stimulated SIRC caused a significant reduction in PGE_2_ levels.

Several findings have been highlighted the role of gabapentin in reducing inflammation in several experimental paradigms (Dias et al., 2014). Recently, Dias et al. (2014) demonstrated that gabapentin decreases the paw edema induced by carrageenan, dextran, and 48/80 in mice. Furthermore, these authors showed that gabapentin inhibited levels of pro-inflammatory cytokines (TNF-α and IL-1β) and neutrophil infiltration. We showed, for the first time, that gabapentin attenuates ocular inflammation elicited by LPS both rabbit corneal cells culture and in rabbit eye. The effects of gabapentin may be due, at least in part, to the inhibition of inflammatory cytokines such as TNF-α, IL-6, and IL-1β. We also demonstrated that tear TNF-α was inhibited by gabapentin in EIU model. The data on TNF-α, IL-6, and IL-1β are in accordance with the findings generated by Lee et al. (2013) in different inflammatory paradigms. The anti-inflammatory effects of gabapentin may be dependent on a combination of pharmacologic properties of this molecule that could be due to the block of some specific cytokines, particularly TNF-α. Similarly to what we observed in SIRC we showed a significant (*p* < 0.05) inhibition of PGE_2_ levels in aqueous humor of rabbit with EIU. It is noteworthy that TNF-α is key actor in ocular inflammation, TNF-α triggers activation of cPLA_2_ and then enhancing the synthesis of PGE_2_ (Van Putten et al., 2001). We demonstrated that ocular inflammation elicited by LPS was significantly attenuated by gabapentin treatment both in SIRC and rabbit eye. TNF-α, a well-known hallmark of ocular inflammation, was significantly reduced by gabapentin treatment *in vitro* and *in vivo* after endotoxin challenging. In particular, gabapentin reduced TNF-α levels in tears, aqueous, cornea, and iris-ciliary body of rabbit with EIU.

## Conclusion

The results generated in the present study suggest that ophthalmic formulation based on gabapentin may be useful in the treatment of inflammatory conditions associated to ocular pain such as uveitis, and that clinical studies to evaluate this possibility may be warranted.

## Author Contributions

Authors make substantial contributions to idea and design: CA, MO, AF, GL, DR, SP, FD, and CB. Authors make contribution to acquisition of data: CA, MO, AF, GL, and CB. Authors make contribution to statistical analysis and interpretation of data: CA, MO, GL, CB, and CG. Authors participate in drafting the article and revising it critically: CA, GL, DR, FD, and CB.

## Conflict of Interest Statement

Co-authors DR and SP are employees of pharmaceutical companies Sooft and Bioos, respectively. The other authors declare that the research was conducted in the absence of any commercial or financial relationships that could be construed as a potential conflict of interest.
